# Culture or Compensation? Trade-Offs Between Organizational Culture Types and Financial Incentives in Romanian Generation Z’s Career Choices

**DOI:** 10.3390/bs16060880

**Published:** 2026-06-01

**Authors:** Vlad Diaconescu, Andreea Fortuna Schiopu, Claudia-Elena Tuclea, Gabriela Tigu, Delia Popescu

**Affiliations:** 1 Doctoral School Business Administration 1, Bucharest University of Economic Studies (ASE), 010374 Bucharest, Romania; diaconescuvlad17@stud.ase.ro; 2 Department of Tourism and Geography, Bucharest University of Economic Studies (ASE), 010404 Bucharest, Romania; andreea.schiopu@com.ase.ro (A.F.S.); gabriela.tigu@ase.ro (G.T.); delia.popescu@com.ase.ro (D.P.)

**Keywords:** organizational culture preferences, cultural fit, scenario-based choice experiments, Competing Values Framework, compensation trade-offs

## Abstract

This study examines trade-offs between organizational culture, measured by the Competing Values Framework, and high financial compensation among Romanian Generation Z (*n* = 584). Using a quantitative cross-sectional design and scenario-based choice experiments, binary logistic regression models assessed how culture preferences and demographics predict employer choice. Results show that preferences for Clan culture most consistently increased the likelihood of selecting a culturally defined employer over high-salary alternatives (odds ratios 1.08–1.15), particularly through mentoring and employee development. Notably, Hierarchy culture preferences also significantly influenced decisions, indicating that this generation values both support and structured predictability. Employment status strongly moderated these trade-offs, with employed respondents significantly more likely to prioritize cultural attributes (odds ratios 2.07–2.47). Findings indicate that Generation Z makes nuanced, context-sensitive trade-offs, challenging one-dimensional assumptions about their motivations. These findings provide context-specific insights into Romanian Generation Z employer choice and offer practical implications for employer branding and talent attraction strategies.

## 1. Introduction

Employer choice typically involves a deliberate trade-off between financial rewards and alignment with the work environment. Substantial evidence links organizational culture to outcomes such as job satisfaction, engagement, and performance, beyond the effects of perceived climate (Hartnell et al., 2011, 2019). A central framework for theorizing culture is Cameron and Quinn’s Competing Values Framework (CVF), which delineates four archetypes: Clan (cohesion and mentoring), Adhocracy (innovation and risk-taking), Hierarchy (structure and control), and Market (competition and results). The CVF is widely used to diagnose culture and guide organizational change (Cameron & Quinn, 2011).

Large-scale longitudinal evidence documents generational shifts in work values, including changes in the relative importance of extrinsic and intrinsic motives among younger cohorts (Twenge et al., 2010). Contemporary global surveys show that Generation Z (Gen Z) considers a “trifecta” of money, meaning, and well-being when making career decisions (Deloitte, 2023), while scholarship reviews highlight the cohort’s growing presence and distinctiveness in the workforce (Benítez-Márquez et al., 2022). Compared with previous cohorts, who often prioritized job security and pay, Gen Z tends to balance financial stability with purposeful work, learning, and quality of life, often through a technology-first, pragmatic approach to employers and work design (Cubukcu Cerasi & Balcioglu, 2024; Zahra et al., 2025). Country-level studies further show how these expectations play out in specific labor markets (e.g., Romania) and how young candidates interpret cultural cues (Diaconescu, 2019).

When cultural fit and salary are compared, experimental evidence indicates that candidates make structured trade-offs among flexibility, advancement prospects, employer brand, and compensation (Styśko-Kunkowska & Kwinta, 2020; Jost & Möser, 2023). Yet these trade-offs are context-dependent and may vary across demographics and task designs, with collegial climate and career prospects often emerging as pivotal attributes. Despite extensive CVF theorizing and the rapidly growing Gen Z literature, we still know relatively little about how Gen Z evaluates specific CVF culture types against a high-salary alternative at the point of choice. This gap matters given Gen Z’s exposure to economic shocks, the pandemic, and platform-based work, which may recalibrate expectations about employer reciprocity and cultural authenticity (Benítez-Márquez et al., 2022; Deloitte, 2023).

This study addresses this gap by employing a scenario-based design grounded in the CVF to examine how Gen Z individuals choose between companies exemplifying Clan, Adhocracy, Hierarchy, or Market cultures and a high-salary alternative. Given the exploratory nature of the scenario-based design and the limited prior evidence on CVF-based salary–culture trade-offs among Romanian Generation Z respondents, this study is guided by research questions rather than directional hypotheses. Specifically, we ask: RQ1. To what extent do preferences for the four CVF culture types predict the choice of a culturally well-defined employer over a higher-pay alternative? RQ2. Which culture type has the strongest predictive power once financial incentives and demographics are considered? RQ3. How do gender, age, residence, income, and employment status influence the relationship between culture preferences and employer choice?

By integrating validated culture theory (CVF), meta-analytic evidence on culture–outcome links, and experimental findings on job-attribute trade-offs, this study contributes to the literature on individual-level career choice by offering exploratory evidence on how CVF-based cultural preferences are reflected in employer choice under salary-culture trade-off conditions.

## 2. Materials and Methods

### 2.1. Literature Review

#### 2.1.1. Theoretical Foundation: The Competing Values Framework

The Competing Values Framework (CVF), developed by Cameron and Quinn (2011), is one of the most widely used and empirically supported approaches to organizational culture. It is based on two competing value dimensions: flexibility versus stability and internal versus external orientation, which combine to form four cultural archetypes (Yu & Wu, 2009). Because these dimensions place opposing assumptions at each end of a continuum, the CVF captures the paradoxical tensions organizations must balance. Evidence from Generation Z business students suggests these distinctions are also behaviorally meaningful: young entrants actively read and evaluate such cues in employer choice, underscoring the importance of cultural fit in early career preferences (Diaconescu, 2024).

Clan culture emphasizes teamwork, empowerment, and a family-like environment, prioritizing collaboration, participation, and consensus; it highlights cohesion, development, and satisfaction over purely financial goals (Cameron & Quinn, 2011). Adhocracy culture centers on innovation, risk-taking, and adaptability, encouraging experimentation, initiative, and entrepreneurial behavior, with success defined by novel outputs. Hierarchy culture relies on formal structures, clear procedures, stability, and control to deliver efficiency, reliability, and predictability (Yu & Wu, 2009). Market culture combines external orientation with stability, stressing competitiveness and customer focus, with success reflected in market share, customer satisfaction, and measurable targets.

The CVF remains influential because it clarifies core design tensions and translates readily into practice across industries. Zeb et al. (2021) show it can guide culture transformation and predict organizational outcomes across performance metrics. Gong et al. (2022) further support its use in culture change by linking each culture type to key resistance forces and matching interventions to transformation scenarios. Adding an individual-level perspective, Diaconescu (2024) shows how Gen Z integrates cultural signals, alongside compensation, into employer evaluations.

#### 2.1.2. Generation Z: Characteristics and Workplace Expectations

Generation Z brings distinctive workplace characteristics compared with earlier cohorts. Despite predictions of post-Great Recession risk aversion, this cohort demonstrates entrepreneurial tendencies, competitiveness, and career aspirations that prioritize growth and learning (Cubukcu Cerasi & Balcioglu, 2024). Evidence also suggests that work is central to their identity (97%), while many describe themselves as job hoppers (83%), seeking diverse skills and value-congruent environments.

Gen Z places strong emphasis on diversity, equity, and inclusion: 88% expect employers to ask about preferred pronouns, and over 40% report willingness to address workplace discrimination, compared with 24% of earlier generations (Zahra et al., 2025). Work–life balance is similarly prioritized (77%), while only 6% cite reaching leadership roles as their primary goal. Financial security remains salient, with higher salary expectations alongside reports of living paycheck to paycheck, indicating a complex link between financial needs and satisfaction (Benítez-Márquez et al., 2022). The most cited motivators are purpose-driven work, competitive compensation, and opportunities for skill development and advancement.

Mental health and well-being are also central, with stress and burnout shaping mobility and employer selection (Diaconescu, 2019). In the context of experienced instability and weaker norms of employer loyalty, Gen Z tends to approach employment pragmatically, emphasizing mutual value creation. Consistent with this, Zahra et al. (2025) find that internal drivers such as enthusiasm and accomplishment are strong motivators, underscoring the role of job-related conditions in sustaining engagement.

#### 2.1.3. Compensation Versus Cultural Factors in Career Decision-Making

The relationship between financial compensation and organizational culture in employee attraction and retention has shifted in contemporary labor markets. Survey evidence shows that over 77% of adults across several countries consider company culture before applying, while 79% prioritize company mission and purpose in job decisions (Wang & Abu Hasan, 2024). This pattern is especially evident among millennials and Generation Z, with many ranking culture above salary.

Aligning compensation systems with organizational culture has therefore become a strategic priority, as pay practices serve as behavioral incentives that shape norms and engagement (Madhani, 2014). When alignment is strong, culture becomes a competitive asset; misalignment can produce unintended effects and reduce effectiveness. Madhani (2014) argues that compensation can strengthen or undermine culture depending on how it is designed, developed, communicated, and managed.

Experimental work by Li and Roloff (2008) shows that compensation structures (merit-based vs. seniority-based) also signal different cultural meanings, shaping applicant attraction and perceived fit. In their study, merit-based systems were associated with perceptions of greater aggressiveness and reward orientation, and lower decisiveness compared with seniority-based systems. Notably, organizations using merit pay were perceived as offering more relational and less transactional psychological contracts than those relying on seniority-based systems.

Recent compensation approaches emphasize transparency, equity, and value alignment, treating pay as both a recruitment tool and a mechanism for reinforcing culture (Wang & Abu Hasan, 2024). Consistent with this broader shift toward non-financial drivers, evidence also shows that intrinsic and social motivations-and related employability resources such as digital skills-strengthen perceived job attractiveness and career intentions (Țuclea et al., 2025). The interaction between organizational culture, leadership behavior, and job satisfaction creates complex dynamics that influence retention and performance. Tsai (2011) found that organizational culture, leadership behavior, and compensation all significantly affect job satisfaction, with organizational culture providing a foundational context for employee attitudes and behaviors.

#### 2.1.4. Research Gaps and Study Contribution

The literature review was structured around three research streams directly relevant to the purpose of the study: CVF-based organizational culture, Generation Z workplace expectations, and salary-culture trade-offs. Despite extensive research on organizational culture typologies and generational workplace preferences, significant gaps remain in understanding how Generation Z weighs cultural factors against financial compensation in career decisions. Although the Competing Values Framework has been validated in many contexts, few studies examine generational responses to specific culture types, particularly among digital natives who are becoming the workforce majority (Yu & Wu, 2009). Research acknowledges that Gen Z seeks a balance between money, meaning, and well-being, yet evidence is limited on how these priorities influence actual choices (Benítez-Márquez et al., 2022), and experimental work on culture–salary trade-offs remains scarce.

This study addresses these gaps using a scenario-based methodology that compares employers representing CVF culture types with high-salary alternatives. The design provides evidence on the relative importance of cultural attributes in career decision-making and identifies demographic and attitudinal predictors of these preferences. By combining established culture theory with current insights into generational expectations, the study contributes to both theory and talent management practice. It also extends CVF application to individual-level career choice behavior, clarifying how cultural typologies shape decision processes and which attributes resonate most with emerging workforce segments. Finally, it responds to Zahra et al.’s (2025) call for research on cross-cultural differences and the role of organizational culture in Gen Z workplace preferences and career decisions.

### 2.2. Methodology

This study employed a quantitative, cross-sectional design with scenario-based choice experiments to examine Romanian Generation Z preferences for organizational culture types relative to high financial compensation. A within-subjects design was used, with participants evaluating multiple hypothetical employment scenarios, allowing direct comparisons across culture dimensions while controlling for individual differences (Cameron & Quinn, 2011).

The study was based on the Competing Values Framework (CVF), which classifies organizational culture into four validated types—Clan, Adhocracy, Hierarchy, and Market—and captures core tensions in organizational design (Cameron & Quinn, 2011). The questionnaire items were developed by adapting the descriptive logic of the CVF/OCAI culture dimensions to an individual preference and employer-choice context. The target population consisted of Generation Z individuals aged 18–26 (born 1997–2005) who were seeking employment or currently employed; participants were required to be at least 18 years old (Benítez-Márquez et al., 2022).

Participants were recruited in Romania through convenience sampling, mainly through online channels, including university-related networks, social media distribution, and direct sharing among eligible young adults. No restrictions regarding industry or educational background were imposed to preserve variability in career stage and professional orientation within the target group. Because the sample was recruited through convenience sampling and included only Romanian respondents, the findings should not be interpreted as representative of Generation Z as a whole. Rather, they provide evidence specific to Romanian young adults included in this sample.

Data were collected using a structured questionnaire that included scenario-based choice experiments and demographic questions. Section I (Culture versus Compensation Trade-offs) presented 24 scenarios in which respondents chose between Company A—offering a “high salary that exceeds even the most optimistic expectations” with no information on climate, management, or colleagues—and Company B, described through culture cues. This formulation was intentionally used to create a strong contrast between financial compensation and cultural attributes. However, because the phrase may have been interpreted differently depending on respondents’ income level, employment status, and expectations, the “high salary” condition should be understood as a perceived high-compensation stimulus rather than as a standardized monetary amount. Scenarios were organized by CVF type: Clan (C1–C6: family-like atmosphere, mentoring, loyalty, human resource development, teamwork, concern for people), Adhocracy (A1–A6: dynamism, innovation, risk-taking, growth, unique outputs, individual initiative), Hierarchy (H1–H6: structure, formal procedures, coordination, efficiency, stability, predictability), and Market (M1–M6: results orientation, competitiveness, demanding management, winning, measurable goals, market share). Section II (direct culture comparisons) included six salary-free pairings: CA, CH, CM, AH, AM, and HM. The direct culture-comparison scenarios collected in Section II were not included in the present analysis because this article focuses specifically on salary–culture trade-offs rather than culture-versus-culture comparisons. These data may be examined in future analyses.

The questionnaire also collected data on gender, age, geographic origin, monthly net income, and employment status (see Table 1).

We further note that both the CVF culture preference indices and the scenario descriptors share the same theoretical origin in the Competing Values Framework (Cameron & Quinn, 2011). As a result, some conceptual overlap exists between the cultural traits measured by the scales and the cultural features depicted in the scenarios. Several design features mitigate the risk of tautological prediction. Scenarios were deliberately constructed to represent CVF culture types, aligning the cultural characteristics of company B in each scenario with the corresponding CVF scale, a necessary step when using both predictors and criteria from the same theoretical typology. This alignment improves face validity. Additionally, the dependent variable is not a cultural preference rating but a forced binary choice between company A and B embedded in a real trade-off between salary and organizational culture, which engages a distinct decision-making cognitive process rather than simply rating a construct. Moreover, this alignment replicates a broader structural property of the CVF instrument: Hartnell et al. (2011) demonstrated meta-analytically that CVF culture types are positively intercorrelated across 84 empirical studies, indicating that the four cultural dimensions share substantial common variance rather than representing mutually exclusive preferences. Nevertheless, the circular element cannot be entirely dismissed. The observed associations may partly reflect participants’ cognitive consistency in affirming culturally congruent attributes across formats, in addition to genuine predictive relationships.

Data were collected through an online survey platform in May 2023. Anonymity and confidentiality were ensured in accordance with applicable data protection legislation, and participants were informed of these safeguards before starting the questionnaire. The survey began with instructions clarifying the scenario-based task: respondents were asked to imagine being accepted by two companies with limited information and to decide solely based on the descriptions provided. Scenarios were displayed one at a time, requiring a forced-choice selection of the preferred company. Completion time was approximately 15–20 min to limit response fatigue while covering all scenario types. To ensure comparability, scenarios were presented in a standardized order across participants.

Data analysis was conducted using SPSS 31 statistical software, employing several analytical approaches:

*Descriptive Analysis*. Frequency distributions and percentages were calculated for each scenario to identify overall preference patterns. Cross-tabulations were used to examine the distribution of choices across demographic variables.

*Reliability Analysis*. Cronbach’s alpha coefficients were calculated for each culture type scale to assess internal consistency reliability. The four culture dimensions (Clan, Adhocracy, Hierarchy, Market) were treated as composite scales, each comprising six items.

*Summative Indices*. Summative scores were created for each culture type by aggregating responses across the six scenarios representing each CVF dimension. These indices enabled quantitative analysis of the strength of culture preferences.

*Logistic Regression Analysis*. Binary logistic regression models were developed to predict the likelihood of choosing Company B (culture-described) over Company A (high salary) for each scenario. The regression equation was specified as:

$$\ln\;(\frac{p}{1-p})=\beta_{0}+\beta_{1}\times \mathrm{Clan}+\beta_{2}\times \mathrm{Adhocracy}+\beta_{3}\times \mathrm{Hierarchy}+\beta_{4}\times \mathrm{Market}+\beta_{5}\times \mathrm{Gender}+\beta_{6}\times \mathrm{Age}+\beta_{7}\times \mathrm{Residence}+\beta_{8}\times \mathrm{Income}+\beta_{9}\times \mathrm{Employment}+u_{0i}.$$

where p represents the probability of choosing Company B, and predictors include preferences for the four culture types along with demographic control variables. 

Model fit was assessed using the following: Omnibus Test of Model Coefficients (Chi-square test evaluating overall model significance); Hosmer–Lemeshow Test (goodness-of-fit assessment for logistic regression models); pseudo R-squared measures (Cox & Snell R^2^ and Nagelkerke R^2^ to assess explained variance); and classification accuracy (percentage of correctly classified cases). Statistical significance was evaluated at the α = 0.05 level. Odds ratios and 95% confidence intervals were calculated to interpret the magnitude and direction of relationships between predictors and outcome variables.

The analytical approach enabled a comprehensive examination of overall preference patterns and individual factors influencing career choice decisions, providing insights into the relative importance of organizational culture characteristics compared to financial compensation among Generation Z job seekers.

## 3. Results

The data were analyzed using several statistical techniques, including frequencies to assess preferences for a specific culture, Cronbach’s alpha to evaluate the reliability of the scales measuring preference for a specific culture, and logistic regression to describe and predict the relationship between the choice of a company offering a specific type of culture.

As shown in Table 2, the measurement scales exhibit strong internal consistency, with Cronbach’s alpha values exceeding the 0.70 threshold. Cronbach’s alpha was used to assess internal consistency, but no factor-analytic validation was conducted in the present study. Therefore, the indices should be interpreted as internally consistent preference indicators rather than fully validated psychometric scales. Based on these items, we computed a summative index for each culture type by aggregating the six items within each construct. Participants then evaluated scenario-based choice tasks and selected the employer they would prefer to work for (Company A or Company B). In Section I, Company A was described only as offering a “high salary that exceeds even the most optimistic expectations,” with no information about work climate, management style, colleagues, or other aspects. Company B was described using culture-specific cues across multiple scenarios, and respondents chose either Company A (high salary) or Company B (a specific work environment).

We analyzed the choice for company B across these scenarios using a logistic regression model specified by the equation:

$$\ln\;(\frac{p}{1-p})=\beta_{0}+\beta_{1}\times \mathrm{Clan culture}\;(C)+\beta_{2}\times \mathrm{Adhocratic culture}\;(A)+\beta_{3}\times \mathrm{Hierarchical culture}\;(H)+\beta_{4}\times \mathrm{Market culture}\;(M)+\beta_{5}\times \mathrm{Gender}+\beta_{6}\times \mathrm{Age}+\beta_{7}\times \mathrm{Residency}+\beta_{8}\times \mathrm{Income}+\beta_{9}\times \mathrm{Employment}+u_{0i},$$

where p is the probability of choosing company B. Predictors included respondents’ preferences for the four culture types, their gender, age, residency, income and employment status.

The logistic regression models were used for exploratory and associative purposes. Given the cross-sectional design and simultaneous measurement of culture preferences and scenario choices, the models should not be interpreted as establishing causal effects.

Because all variables were collected based on self-reported data and at the same time, common method bias might be a concern in this study. We present herein several methodological and statistical arguments to demonstrate that this risk has been mitigated. First, since the dependent variable (employer preferences) uses a forced-choice scenario selection (nominal scale) and independent variables (cultural preferences) use Likert CVF scales, common method bias is less likely to inflate the present results. This methodological heterogeneity decreases the probability that response consistency alone drives the observed associations (Podsakoff et al., 2003). Second, the scenario-based design confronts respondents with a decision-based context, asking them to choose between a high salary and a specific type of cultural environment, which involves a distinct cognitive process from simply rating cultural preferences. Third, we conducted Harman’s single-factor test on all 24 CVF items. The first unrotated factor accounted for 45.01% of the total variance, which is below the 50% threshold commonly cited in the common method bias literature (Podsakoff et al., 2003). This test provides preliminary evidence that common method variance does not substantially distort the relationships among the studied variables. However, it should be considered only a supplementary and illustrative check, not definitive evidence against common method bias. The stronger safeguards in this study are the use of different measurement formats and the scenario-based forced-choice design.

Prior to the main analysis, we also assess the potential multicollinearity among the CVF culture preference scales as seen in Table 3, examining the correlation matrix of the four predictors and computed Variance Inflation Factors (VIF) for each. The four summative indices exhibit moderate to high positive intercorrelations (r = 0.540 − 0.755, *p* < 0.001), a pattern consistent with meta-analytic findings reported by Hartnell et al. (2011) across 84 empirical studies using the CVF instrument (average r = 0.54). VIF values remain below conventional thresholds cited by Hair et al. (2010) (Clan: VIF = 2.21; Adhocratic: VIF = 2.86; Hierarchical: VIF = 2.86; Market: VIF = 2.50), indicating that multicollinearity does not distort the regression estimates.

As shown in Table 4, respondents preferred Company B in all Clan scenarios, even though Company A offered a high salary, with a majority choosing Company B each time. This pattern suggests that young adults value an open and friendly work environment, mentoring-oriented managers, group loyalty and tradition, long-term human resource development, teamwork and consensus, and organizational concern for people. The strongest preference for Company B was in scenario C6 (“strong concern for people”), where 74.1% selected Company B over the high-salary alternative. To examine which factors predict these choices, we next estimate scenario-specific logistic regression models that include the culture preference indices and the demographic variables described above.

The results in Table 5 show that the logistic regression models have an acceptable fit and can be used for predictive interpretation across the Clan scenarios. For scenario C1 (open and friendly workplace; “big family”), the model is statistically significant (Omnibus Test: χ^2^(15) = 48.567, *p* < 0.001), indicating that the set of predictors improves classification of the choice between Company A and Company B compared to the null model. The Hosmer–Lemeshow test is non-significant (χ^2^(8) = 10.360, *p* = 0.241), suggesting adequate model fit. The model explains 11.2% of the variance in the choice outcome (Nagelkerke R^2^ = 0.112) and correctly classifies 70.9% of cases. The relatively modest Nagelkerke R^2^ values indicate that cultural preferences and demographics explain only part of employer choice. This is expected given the complexity of career decision-making, which may also depend on unmeasured factors such as career goals, industry preferences, perceived employability, family obligations, or risk tolerance. The remaining Clan scenario models (C2–C6) are also statistically significant (Omnibus Test, *p* < 0.05) and show adequate fit (Hosmer–Lemeshow *p* > 0.05). Their explanatory power ranges from 8.3% (C4) to 12.5% (C6) (Nagelkerke R^2^ = 0.108 for C2, 0.107 for C3, 0.083 for C4, 0.118 for C5, and 0.125 for C6). We next examine the significant predictors for each scenario (Table 6); for clarity, only statistically significant coefficients are reported.

In scenario C1 (open and friendly “big family” environment), the likelihood of choosing Company B over the high-salary Company A is significantly predicted by preferences for Clan and Market culture. A one-unit increase in Clan culture preference increases the odds of choosing Company B by 14% (OR = 1.14, *p* < 0.001), while a one-unit increase in Market culture preference decreases the odds by 7% (OR = 0.93, *p* = 0.010). The corresponding model is:

$$\ln\;(\frac{p}{1-p})=0.745+0.129\times \mathrm{Clan culture}\;(C)-0.078\times \mathrm{Market culture}\;(M),$$

where p is the probability of choosing Company B.

For scenario C2 (managers as mentors/parental figures), significant predictors include Clan culture preference (OR = 1.11, *p* < 0.001), Hierarchy culture preference (OR = 1.08, *p* = 0.012), and employment status. Employed respondents are more likely to choose Company B (OR = 2.07, *p* = 0.016). In scenario C3 (group loyalty and tradition), Clan preference again increases the odds of choosing Company B (OR = 1.13, *p* < 0.001), while Adhocracy preference slightly decreases them (OR = 0.94, *p* = 0.035). Hierarchy preference is also positive (OR = 1.08, *p* = 0.012), and employed respondents have higher odds of choosing Company B (OR = 2.16, *p* = 0.009), representing a 116% increase in odds relative to the reference category. In scenario C4 (long-term benefits of human resource development), Hierarchy preference is the key predictor (OR = 1.11, *p* < 0.001). Clan preference is marginal (OR = 1.06, *p* = 0.051), just above the conventional 0.05 threshold. In scenario C5 (teamwork, participation, and consensus), both Clan (OR = 1.08, *p* = 0.007) and Hierarchy preferences (OR = 1.09, *p* = 0.003) significantly increase the odds of choosing Company B. Gender is also significant (OR = 1.45, *p* = 0.046), with male respondents more likely to select Company B. In scenario C6 (strong concern for people), only Clan preference significantly predicts choosing Company B (OR = 1.15, *p* < 0.001).

Overall, across Clan scenarios, preference for Clan culture is the most consistent predictor of choosing the culturally described employer over the high-salary alternative. Hierarchy preference, employment status, and gender show additional scenario-specific effects.

As shown in Table 7, the second set of scenarios focuses on Adhocracy culture (A1–A6), where Company B is described with cues such as a dynamic, entrepreneurial, and creative workplace (A1); acceptance of innovation and risk-taking (A2); openness to new and different thinking (A3); long-term growth and resource acquisition (A4); success defined by unique products or services and sector leadership (A5); and encouragement of individual initiative and freedom (A6), each compared to Company A, which offers a high salary. Respondents preferred Company B in five of the six scenarios, particularly in A1, A3, and A6. The exception was A5, where a majority selected the high-salary option, suggesting that “being a sector leader” is not, by itself, sufficiently attractive when salary is salient. Scenario A6 shows the strongest preference for Company B, with over 70% choosing the option emphasizing individual initiative and freedom.

Logistic regression was again used to model the likelihood of choosing Company B across the Adhocracy scenarios (A1–A6). As shown in Table 8, all six models are statistically significant (Omnibus Test: *p* < 0.05) and show adequate fit, with non-significant Hosmer–Lemeshow tests (*p* > 0.05) in each case. Explanatory power is modest, ranging from 6.0% in A3 to 11.5% in A4 (Nagelkerke R^2^ = 0.060 and 0.115, respectively). Classification accuracy ranges from 60.8% (A2) to 72.9% (A6), indicating consistent predictive performance across scenarios.

Table 9 presents the significant predictors of choosing Company B (Adhocracy scenarios A1–A6) over the high-salary Company A. Overall, preference for Adhocracy culture is a consistent positive predictor, along with scenario-specific effects of Hierarchy preference, employment status, and income. For example, in A1 (dynamic, entrepreneurial, and creative workplace), both Adhocracy preference and employment status category 3 (not working and never employed) are significant. A one-unit increase in Adhocracy preference increases the odds of choosing Company B by 11% (OR = 1.11, *p* = 0.002), and respondents in employment category 3 have higher odds of selecting Company B (OR = 2.38, *p* = 0.043). Across scenarios, Adhocracy preference increases the odds of choosing Company B (ORs ≈ 1.07–1.11; *p* < 0.05), indicating that stronger endorsement of dynamic, innovative, and initiative-oriented work environments is associated with greater preference for the culturally described option.

Income is significant in A4 and A5. In A4 (growth and resource acquisition), lower income (category 1) reduces the odds of choosing Company B (OR = 0.59, *p* = 0.050), while employment category 3 increases the odds (OR = 2.47, *p* = 0.015). In A5 (unique products/services and sector leadership), preference for Hierarchy culture and higher income (category 3) predict choosing Company B; higher-income respondents have 2.23 times greater odds of selecting Company B (OR = 2.23, *p* = 0.021).

Employment status shows a recurring effect in this set: respondents in category 3 are more likely to choose Company B in A1, A4, and A6 (ORs > 2; *p* < 0.05), with a marginal effect in A3 (*p* = 0.055). This pattern may suggest that respondents without work experience are particularly attracted to Adhocracy cues such as dynamism, innovation, and flexibility, while the high-salary alternative may be perceived as carrying greater pressure or less appealing contextual characteristics.

As shown in Table 10, the third set of scenarios focuses on Hierarchy culture (H1–H6), where Company B is described with cues such as a highly structured and formal workplace governed by rules and procedures (H1), efficiency-oriented coordinators and organizers (H2), formal policies holding the group together (H3), long-term goals of stability and efficient operations (H4), success defined by reliability, smooth scheduling, and low costs (H5), and management emphasis on security and predictability (H6), each compared with Company A offering a high salary. Overall, preferences are mixed: respondents choose the high-salary option in three scenarios (H1, H3, and H6), indicating that strongly formalized and rule-bound environments and an emphasis on predictability are less attractive to many Gen Z respondents. In contrast, Company B is preferred in H2, H4, and H5 (61.8%, 57.7%, and 56.0%, respectively), suggesting that coordination, efficiency, and operational stability can outweigh salary when presented in less restrictive terms. To examine which factors predict these choices, we next estimate scenario-specific logistic regression models including the culture preference indices and demographic variables (gender, age, residency, income, and employment status).

We then estimated logistic regression models for the Hierarchy scenarios (H1–H6). As shown in Table 11, model fit is acceptable across all scenarios, with non-significant Hosmer–Lemeshow tests (*p* > 0.05) in each case. Five models are statistically significant (Omnibus Test, *p* < 0.05), while H5 does not reach conventional significance (*p* = 0.086) and should be interpreted with caution. Explanatory power is modest, ranging from 5.1% (H5) to 13.3% (H1) (Nagelkerke R^2^ = 0.133 for H1, 0.102 for H2, 0.0124 for H3, 0.060 for H4, 0.051 for H5, and 0.093 for H6). Classification accuracy ranges from 60.1% (H4) to 65.9% (H1).

Predictor estimates (Table 12) indicate that the Hierarchy culture preference score is a significant positive predictor in all six scenarios (H1–H6), showing that respondents who value hierarchical culture are consistently more likely to choose Company B over the high-salary alternative. Gender (category 1: males) is significant in two scenarios (H2 and H4), suggesting a gender-specific effect for preferences related to coordination and efficiency, and long-term stability and operations.

Hierarchical culture preference is a significant positive predictor in all six scenarios (Table 11). A one-unit increase in the Hierarchy score increases the odds of choosing Company B by 19% in H1 (OR = 1.19, *p* < 0.001) and by 15% in H2 (OR = 1.15, *p* < 0.001). Similar effects are found in H3 (OR = 1.16, *p* < 0.001), H4 (OR = 1.07, *p* = 0.016), H5 (OR = 1.09, *p* = 0.003), and H6 (OR = 1.16, *p* < 0.001). Overall, odds ratios range from 1.07 to 1.19 (all *p* < 0.05), indicating a consistent preference for the culturally defined employer among respondents who value hierarchical features. Adhocracy preference shows a small negative effect in H3 (OR = 0.93), suggesting that stronger endorsement of adhocratic values slightly reduces preference for Company B when formal policies are emphasized.

Gender (category 1: males) is significant in H2 and H4. Male respondents have higher odds of choosing Company B when it is described as efficiency-oriented and well-coordinated (H2: OR = 1.61, *p* = 0.011) and when it emphasizes long-term stability and efficient operations (H4: OR = 1.53, *p* = 0.019). This pattern suggests that, within this sample, men are relatively more attracted to organizational cues related to coordination, structure, and operational stability.

As shown in Table 13, the Market culture scenarios (M1–M6) produce a split pattern, with respondents relatively evenly divided between Company A (high salary) and Company B (market-oriented cues). Company B is slightly preferred in M1, M3, and M4, where it is described as results-oriented and productive, led by demanding and persistent managers, and unified by a focus on winning and shared concerns for reputation and success. In contrast, a slight majority prefer the high-salary option in M2 (competitive, goal-oriented people) and M5 (long-term emphasis on competitive action and measurable targets), while a clear majority choose Company A in M6 (high market share and competitive prices). This suggests that some performance-driven cues can outweigh salary, whereas market dominance signals (market share and competitive pricing) are not necessarily interpreted as direct employee benefits.

To examine which factors predict these choices, we estimated scenario-specific logistic regression models (Table 14 and Table 15). As shown in Table 14, all six Market models are statistically significant (Omnibus Tests, *p* < 0.001) and show adequate fit, with non-significant Hosmer–Lemeshow tests (*p* > 0.05) in each case. Explanatory power is modest: the models explain 8.6–12.7% of the variance in choosing Company B over Company A (Nagelkerke R^2^ = 0.096 for M1, 0.127 for M2, 0.127 for M3, 0.087 for M4, 0.119 for M5, and 0.086 for M6). Classification accuracy ranges from 60.6% (M4) to 64.4% (M2).

Based on Table 15, hierarchy culture preference is a consistent positive predictor of choosing Company B across all market scenarios (M1–M6), with odds ratios ranging from 1.08 to 1.14 (all *p* < 0.05). This indicates that respondents who value hierarchical features are more likely to select the culturally described employer even when scenarios emphasize market-oriented cues. In contrast, adhocracy preference is negatively associated with choosing Company B in M2 and M3 (OR = 0.93 in both cases), suggesting that stronger endorsement of adhocratic values slightly reduces preference for Company B when competitiveness or demanding productivity cues are highlighted. Employment status is significant in M3 (demanding, persistent, productive managers): both employed respondents (category 1) and those not employed with no work experience (category 3) show higher odds of choosing Company B (OR = 2.31 and OR = 2.09, respectively; both *p* < 0.05). Gender is also significant in M4, where males (category 1) are more likely to choose Company B (OR = 1.46, *p* = 0.037), indicating a gender effect for scenarios centered on winning and shared reputation or success. Finally, in M6, market culture preference is a modest positive predictor (OR = 1.06, *p* = 0.043), and higher income (category 3) substantially increases the odds of choosing Company B (OR = 2.09, *p* = 0.033).

The results presented in Table 15 indicate that cultural perceptions (especially hierarchical culture), employment status, gender, income, and specific cultural dimensions (adhocratic, market) have varying influences on the choice of company B across different scenarios. Overall, for this set of scenarios, hierarchical culture is a key positive influence, while adhocratic culture may reduce preference in certain contexts.

## 4. Discussion

The Romanian context is relevant for interpreting these findings. Romania combines relatively lower wage levels compared to much of the European Union, substantial youth migration, and a post-socialist organizational legacy that may shape attitudes toward hierarchy, stability, and career security differently from Western European contexts. In this environment, Generation Z may draw a clearer distinction between enabling organizational structure (coordination, efficiency, predictability) and constraining bureaucracy (formalism, rigid procedures). This contextual perspective may help explain why hierarchy-related preferences remained consistently significant across several scenarios.

The findings show that Romanian Generation Z respondents do not view salary and organizational culture as separate criteria, but as interrelated elements when choosing an employer. Overall, their decisions reflect context-dependent trade-offs between financial incentives and specific cultural cues.

### 4.1. Key Findings and Comparison with Prior Research

RQ1: Culture Types as Predictors of Employer Choice

The findings indicate that CVF culture preferences are associated with employer choice under salary-culture trade-off conditions, although the strength and direction of these associations vary by culture type. Clan culture is the most consistent predictor, suggesting that relational and developmental cues remain highly salient for Romanian Gen Z respondents even when a high-salary alternative is available. This extends previous evidence on the importance of organizational culture for employee outcomes (Hartnell et al., 2011, 2019) by showing that culture-related preferences may also matter at the pre-employment stage.

The descriptive results support this interpretation. Scenarios emphasizing concern for people, open and friendly workplaces, and human resource development attracted clear majorities, aligning with Deloitte’s (2023) view that Gen Z considers money, meaning, and well-being in career decisions. At the same time, these findings nuance claims that Gen Z’s pragmatic and financially conscious orientation would necessarily lead to prioritizing pay over culture (Benítez-Márquez et al., 2022). Instead, respondents appear to evaluate compensation and culture together.

Hierarchy-related findings are more ambivalent. While Hierarchy preference significantly predicted choosing Company B across the hierarchy scenarios, respondents rejected strongly rule-bound, formalized, or security-focused portrayals in favor of salary. However, they were more receptive to scenarios framed around coordination, efficiency, stability, and reliability. This suggests that Romanian Gen Z respondents distinguish between constraining bureaucracy and enabling structure. Therefore, the results refine Cameron and Quinn’s (2011) framework by showing that concrete manifestations of a culture type may matter more than the archetype label itself. This also suggests that, at the individual preference level, CVF culture types may operate less as mutually exclusive alternatives and more as combinations of valued workplace attributes. Our results indicate medium to high intercorrelations among four CVF culture preference indices, which is consistent with Hartnell et al. (2011) who reported positive correlations among all CVF culture types across 84 empirical studies, contrary to the CVF theory. Hartnell et al. (2011) suggested that CVF culture types may function as complementary rather than contradictory, proposing an alternative theoretical approach to account for the synergistic interactions among the cultural values that define an organization culture. They understand organizational culture as an integrated pattern of values rather than a set of mutually exclusive types. The present findings extend this observation to the individual preference level: participants who score highly on one cultural orientation tend to score positively on others as well, reflecting broad, overlapping value preferences rather than discrete competing dispositions. This configurational approach also partially addresses concerns regarding the conceptual alignment between CVF scale content and proposed scenario descriptors in our study. 

RQ2: Relative Predictive Power of Culture Types

Across the 24 scenarios, Clan culture showed the strongest and most stable predictive role, followed by Hierarchy culture. The repeated significance of Hierarchy preference across several culture sets suggests that structure, predictability, and coordination may serve as cross-cutting preferences rather than as values opposed to relational or developmental concerns. This challenges a strict interpretation of the CVF as a framework of purely competing values, suggesting that, for this sample, supportive environments and clear organizational systems can be complementary. This interpretation is consistent with the empirical pattern in which Clan and Hierarchy preferences jointly predict employer choice, suggesting that support and structure may be complementary rather than contradictory for Romanian Gen Z respondents. The widespread effect of Hierarchy preference may further indicate that respondents interpreted hierarchy-related items less as a preference for bureaucracy and more as a broader preference for clarity, coordination, and predictability. This may explain why Hierarchy preference appears across several non-Hierarchy scenarios and supports a more integrative interpretation of CVF dimensions at the individual preference level. Another direct theoretical explanation for the reach of Hierarchical culture predictive power across scenarios are the strong intercorrelation of this culture type with Market culture (r = 0.755) and Adhocratic culture (r = 0.694) presented in Table 3). Participants who score highly on this dimension tend to also score highly on other dimensions, meaning Hierarchical culture partly captures shared variance common to all four orientations. This finding is consistent with prior research (Hartnell et al., 2011) noting that CVF measures are not always empirically orthogonal in self-report data. Hierarchical culture’s emphasis on structure, stability, and predictability may be seen as a generalized disposition toward organizational formalization that transcends specific cultural boundaries, explaining its consistent positive association with Company B preference across all scenarios. 

Additionally, the predictive power of Hierarchical culture across scenarios indicates that our relationships are not purely tautological; if they were, they would merely replicate the scenario’s implied cultural type. If the observed associations were driven solely by item-level overlap or response consistency, one would expect each culture type to predict only its matched scenarios. This pattern in our results speaks against purely tautological prediction: Hierarchical culture emerges as a significant positive predictor of company B preference across scenarios belonging to the Adhocratic and Market culture sets. This suggests that our models capture theoretically meaningful variance beyond mere response consistency.

Adhocracy produced more conditional results. It positively predicted choices in its own scenarios, particularly where autonomy, innovation, and freedom were emphasized, but showed negative associations in some non-Adhocracy contexts. This suggests that innovation and risk-taking are attractive when framed as opportunities, but less appealing when perceived as potentially undermining stability or predictability. These findings add nuance to prior work on job-attribute trade-offs (Styśko-Kunkowska & Kwinta, 2020) by indicating that trade-offs may also occur among cultural attributes, not only between salary and non-salary factors. This partially contrasts with literature portraying Generation Z as strongly innovation-oriented, suggesting that innovation-related cues may be attractive only when they do not imply instability or excessive uncertainty.

Market culture had the weakest and least consistent role. Its external success cues, such as market share or competitive pricing, did not consistently translate into perceived employee benefits. This supports the interpretation that Romanian Gen Z respondents evaluate employers pragmatically and do not automatically equate organizational competitiveness with an attractive work experience, consistent with Cubukcu Cerasi and Balcioglu’s (2024) observations.

RQ3: Demographic Influences on Culture-Choice Relationships

Employment status emerged as an important moderator. Employed respondents were more likely to choose culturally defined companies in several scenarios, suggesting that work experience may increase awareness of non-financial workplace attributes. In contrast, respondents without work experience appeared more attracted to some Adhocracy cues, especially innovation, autonomy, and freedom. This extends Diaconescu’s (2024) qualitative findings by showing that employment experience shapes how Gen Z interprets cultural signals.

Gender effects were selective rather than general. Male respondents had higher odds of preferring scenarios emphasizing efficiency, stability, and competitive orientation, which is broadly consistent with differentiated job-preference patterns identified by Jost and Möser (2023). However, the absence of gender effects in most Clan scenarios suggests that relational and developmental attributes appeal broadly across genders.

Income effects were limited but meaningful. Higher-income respondents were more likely to choose scenarios emphasizing uniqueness and market leadership, while lower income reduced preference for long-term growth scenarios. These results support Benítez-Márquez et al.’s (2022) observation that financial precarity may limit the extent to which Gen Z can prioritize cultural attributes over financial considerations. The income-related effect observed in A4 should be interpreted with caution because the “high salary” stimulus was intentionally left unanchored and may have represented different subjective gains for respondents. For lower-income participants, the salary alternative may have reflected a much greater perceived improvement in financial security, making it difficult to distinguish between a genuine preference for compensation and a stronger relative attractiveness of the salary stimulus itself. Future studies could improve interpretability by using explicit salary multiples (e.g., 1.5× or 2× local market salary levels) instead of subjective high-salary descriptions. Overall, the findings show that Gen Z should not be treated as a homogeneous cohort: culture–compensation trade-offs vary by employment experience, gender, income, and scenario framing. 

### 4.2. Theoretical Implications

This study offers four theoretical implications. First, it extends the Competing Values Framework (CVF) to individual-level career decision-making, showing that CVF culture preferences are associated with employer choice even when a high-salary alternative is explicit. This supports the framework’s relevance for pre-employment attitudes, beyond its typical organizational-level use. Second, the findings suggest that CVF dimensions are not experienced as strictly competing values in individual preferences. The positive effects of both Clan and Hierarchy preferences indicate that Generation Z may seek a combination of people-oriented support and clear structure, qualifying Cameron and Quinn’s (2011) emphasis on fundamental tensions and pointing to a more integrative preference pattern. Third, the study contributes to generational theory by providing experimental evidence on culture–compensation trade-offs. While consistent with claims that Generation Z values meaning and culture (Deloitte, 2023; Zahra et al., 2025), the results show systematic variation by culture type, employment experience, and demographics, indicating conditional rather than universal “culture over salary” preferences. Fourth, the research contributes to discrete-choice and trade-off scholarship (Styśko-Kunkowska & Kwinta, 2020; Jost & Möser, 2023) by showing that culture typologies can offer predictive leverage when modeling career decisions, with the CVF capturing coherent psychological dimensions that shape choice behavior.

### 4.3. Practical Implications

For organizations seeking to attract and retain Gen Z talent, the findings suggest several practical implications. Clan culture cues-mentoring, concern for people, human resource development, and teamwork-are generally effective, but “family-like” messaging should be used only when supported by credible practices, given Gen Z’s sensitivity to authenticity (Benítez-Márquez et al., 2022). Organizations with hierarchical cultures should present structure as clarity, coordination, efficiency, and reliable systems, rather than as control, rigid rules, or procedures. Adhocracy messaging should be tailored to work experience: innovation and initiative may appeal more to those without employment experience, while employed Gen Z may need these cues balanced with stability and structure. Market culture messaging should explicitly link competitiveness and external success to employee benefits, such as development, rewards, and career opportunities. Finally, culture does not replace pay: competitive compensation remains necessary, but differentiation increasingly depends on authentic culture-employee fit. Recruitment communication should therefore combine relational and developmental signals with credible employee experiences and Gen Z testimonials, consistent with Diaconescu (2019). For talent management and employer branding, this means that organizations should not promote culture as a generic value proposition, but should communicate specific and credible combinations of support, development, structure, and career benefits.

### 4.4. Limitations and Future Research

Several limitations should be noted. First, the scenario-based design captures stated preferences rather than observed behavior. Future studies could strengthen the behavioral validity of the scenario-based design by comparing stated preferences with actual application behavior, job-search data, or longitudinal employment outcomes. In addition, the use of a convenience sample from a single national context limits generalizability; therefore, the findings should be interpreted as context-specific evidence on Romanian Generation Z rather than as universal claims about the cohort. A further limitation concerns the partial conceptual overlap between the culture preference indices and the scenario descriptions. Because both were derived from the same CVF logic, some regression effects may reflect consistency between general culture preferences and scenario-specific evaluations rather than independent prediction. Although several design features reduce the risk of tautological prediction, including the company forced-choice format, the salary trade-off, and the cross-cultural predictive patterns observed in the results, the circular element cannot be entirely dismissed. The observed relationships may partly reflect participants’ cognitive consistency in affirming culturally congruent attributes across the two measurement formats used in this study, in addition to genuine associative relationships. Future studies should consider developing scenario descriptors that are behaviorally anchored and constructed independently of CVF item content, employing expert validation procedures to ensure that scenario stimuli do not reproduce scale language.

Because all variables were collected in the same survey at the same time, common method bias cannot be entirely ruled out, but we presented arguments that the risk has been mitigated. Though, scenario order was not randomized, which represents a limitation that future studies should address. Because Market culture scenarios were always presented after respondents completed the Clan, Adhocracy, and Hierarchy scenarios, the weaker and more divided Market results may partly reflect fatigue or contrast effects rather than substantive differences. Future research should reduce this risk through temporal separation, response randomization, multi-source data, or behavioral validation.

Although experiments strengthen internal validity, field studies of actual job choices would complement these results, and longitudinal designs could test whether pre-employment preferences predict later satisfaction and retention. Second, the “high salary exceeding even most optimistic expectations” framing may have been too abstract for some respondents. Because this formulation was subjective, respondents may have interpreted it differently depending on their income level, employment status, and salary expectations. Company A was intentionally described with limited information in order to isolate the salary cue, whereas Company B contained richer cultural information. This asymmetry may have introduced ambiguity aversion, meaning that some respondents may have preferred Company B partly because it was more fully described rather than solely because of its cultural attributes. Future work could specify salary levels relative to market rates and vary premium sizes to identify tipping points where pay outweighs culture. Third, culture types were presented largely in isolation rather than as mixed organizational profiles; since most organizations combine CVF dimensions (Cameron & Quinn, 2011), testing richer culture configurations would improve external validity. Fourth, the modest explained variance (Nagelkerke R^2^ = 0.051–0.133) indicates that additional drivers of employer choice remain unmeasured. These R^2^ values show that cultural preferences account for a representative but partial portion of the variance in employer preference decisions, which is theoretically coherent. These models are useful to identify which cultural orientations affect the probability of choosing a culturally defined company over a high-salary alternative. This also means that the predictive role of culture preferences should be interpreted cautiously and as only one component of a broader employer-choice process. Future studies should incorporate factors such as career stage, career goals, industry preferences, mobility constraints, and family responsibilities. The use of 24 separate logistic regression models increases the risk of Type I error. We opted against applying a Bonferroni correction for several reasons. First, the 24 models are not independent tests of the same hypothesis, but address different scenarios describing four qualitative cultural environments. We consider inappropriate employing a correction such as Bonferroni in this context, since it assumes a single family of tests sharing one null hypothesis and would create a high rate of Type II errors. Additionally, each scenario represents a distinct research context detailing employer preference under different cultural conditions, and the exploratory nature of the study prioritizes the ability to identify potentially meaningful predictive patterns over strict error rate control. However, given the number of models estimated, the individual significance levels should be interpreted with caution. Fifth, the study did not assess perceived culture–compensation substitutability: some respondents may expect culturally positive employers to offer competitive pay over time, while others may interpret the trade-off as fixed. Manipulating compensation trajectories alongside culture cues would clarify these assumptions.

Future research should explore several promising directions. First, examining how Gen Z evaluates cultural authenticity and organizational hypocrisy when stated culture conflicts with employee reviews or behavioral evidence would inform recruitment ethics. Second, investigating how cultural preferences evolve through career transitions-first job, job changes, career progression-would reveal developmental patterns. Third, studying how organizational culture interacts with remote and hybrid work arrangements, which are increasingly important for Gen Z, would address contemporary work design questions. Fourth, comparing Gen Z cultural preferences with those of older generations in the same organizations would clarify generational effects versus age or career stage effects. Finally, examining how macroeconomic conditions moderate culture–compensation trade-offs would test whether cultural preferences persist during economic downturns versus periods of prosperity.

## 5. Conclusions

This study shows that Romanian Generation Z respondents make nuanced, context-dependent trade-offs between organizational culture and financial compensation. Clan culture cues consistently predict the selection of culturally defined employers, while preferences related to hierarchy depend on how structure is presented. Employment experience, gender, and income further moderate these relationships, confirming meaningful variation within the cohort.

Overall, the findings challenge one-dimensional claims that Generation Z prioritizes either meaning or money. Instead, employer choice appears to be shaped by culture type, personal circumstances, and specific organizational signals. For researchers and practitioners, this underscores the need for more refined models of employer choice that integrate cultural typologies and demographic moderators. Organizations that credibly combine relational and developmental cues, functional structure, and competitive pay are likely to strengthen Generation Z talent attraction and retention.

## Figures and Tables

**Table 1:** Sample Structure.

Demographic Characteristics	Frequency (*n*)	Percentage (%)
**Total sample**	**584**	**100**
**Gender**		
male	272	46.6
female	312	53.4
**Age**		
18–21 years	281	48.1
21–23 years	172	29.5
23–26 years	131	22.4
**Place of origin**		
Bucharest	233	39.9
Another city	246	42.1
Rural area	105	18.0
**Net monthly individual income**		
<1500 RON (~€300)	216	37.0
1500–2500 RON (~€300–€500)	135	23.1
2501–4000 RON (~€500–€800)	141	24.1
>4000 RON (>~€800)	92	15.8
**Employment status**		
Employee	294	50.3
Not currently employed, but previously employed	120	20.5
Never employed	128	21.9
Own/family business	42	7.2

**Table 2:** Measurement instrument (culture types): Reliability analysis.

Culture Category	Cronbach Alpha
Clan culture (C)—6 items	0.872
Adhocratic culture (A)—6 items	0.864
Hierarchical culture (H)—6 items	0.861
Market culture (M)—6 items	0.867

**Table 3:** Correlations among CVF Culture Preference Indices.

Culture Category	Clan Culture (C)	Adhocratic Culture (A)	Hierarchical Culture (H)	Market Culture (M)
Clan culture (C)	-			
Adhocratic culture (A)	0.729 ***	-		
Hierarchical culture (H)	0.598 ***	0.694 ***	-	
Market culture (M)	0.540 ***	0.645 ***	0.755 ***	-
**VIF**	**2.21**	**2.86**	**2.86**	**2.50**

Note: *N* = 584. VIF = Variance Inflation Factor. *** *p* < 0.001.

**Table 4:** Clan culture scenarios: Company choice between A and B.

Scenarios	Company A (High Salary)		Company B (As Described)	
	Frequency	%	Frequency	%
C1. Company B offers an open and friendly workplace, with people being like a big family.	188	32.2	396	67.8
C2. In Company B managers are considered to be mentors or even parental figures (like parents).	227	38.9	357	61.1
C3. In Company B, group loyalty and a sense of tradition are strong.	241	41.3	343	58.7
C4. In Company B, the emphasis is on the long-term benefits of human resource development.	213	36.5	371	63.5
C5. Company B prioritizes teamwork, participation, and consensus, and places great importance on group cohesion.	235	40.2	349	59.8
C6. In Company B there is a strong concern for people.	151	25.9	433	74.1

**Table 5:** Clan culture vs. compensation: Model fit Logistic regression results for different scenarios.

Scenario	Omnibus Test		Hosmer–Lemeshow		−2 Log Likel.	Cox & Snell R^2^	Nagelkerke R^2^	Class. Acc. (%)
	χ^2^ (df)	sig	χ^2^ (df)	sig				
C1. Company B offers an open and friendly workplace, with people being like a big family.	48.567 (15)	<0.001	10.360 (8)	0.241	685.295	0.080	0.112	70.9
C2. In Company B managers are considered to be mentors or even parental figures (like parents).	48.449 (15)	<0.001	5.969 (8)	0.651	731.964	0.080	0.108	65.8
C3. In Company B, group loyalty and a sense of tradition are strong.	48.406 (15)	<0.001	7.161 (8)	0.519	743.283	0.080	0.107	64.4
C4. In Company B, the emphasis is on the long-term benefits of human resource development.	36.410 (15)	0.002	4.574 (8)	0.802	729.902	0.060	0.083	65.8
C5. Company B prioritizes teamwork, participation, and consensus, and places great importance on group cohesion.	53.602 (15)	<0.001	12.023 (8)	0.150	733.597	0.088	0.118	65.9
C6. In Company B there is a strong concern for people.	52.159 (15)	<0.001	9.228 (8)	0.323	615.408	0.085	0.125	73.3

**Table 6:** Clan culture vs. compensation: Logistic Regression Coefficients and Odds Ratios.

Scenario	Predictor	B	S.E.	Wald	*p*-Value	Exp(B)	95% CI for Exp(B)
C1. Company B offers an open and friendly workplace, with people being like a big family.	Constant	0.745	0.089	70.75	<0.001	2.11	
	Clan culture	0.129	0.030	18.19	<0.001	1.14	(1.07; 1.21)
	Market culture	−0.078	0.030	6.60	0.010	0.930	(0.87; 0.98)
C2. In Company B managers are considered to be mentors or even parental figures (like parents).	Constant	−1.346	0.574	5.50	0.019	0.260	
	Clan culture	0.106	0.029	13.68	<0.001	1.11	(1.05; 1.18)
	Hierarchical culture	0.076	0.030	6.36	0.012	1.08	(1.02; 1.15)
	Employment (1)	0.729	0.301	5.86	0.016	2.07	(1.15; 3.74)
C3. In Company B, group loyalty and a sense of tradition are strong.	Constant	−2.297	0.584	15.48	<0.001	0.10	
	Clan culture	0.124	0.029	18.62	<0.001	1.13	(1.07; 1.20)
	Adhocratic culture	−0.066	0.032	4.43	0.035	0.94	(0.88; 0.96)
	Hierarchical culture	0.076	0.030	6.36	0.012	1.08	(1.02; 1.14)
	Employment (1)	0.768	0.295	6.79	0.009	2.16	(1.21; 3.84)
C4. In Company B, the emphasis is on the long-term benefits of human resource development.	Constant	−1.855	0.580	10.21	0.001	0.156	
	Clan culture	0.054	0.028	3.82	0.051	1.06	(1.00; 1.12)
	Hierarchical culture	0.103	0.030	11.66	<0.001	1.11	(1.05; 1.18)
C5. Company B prioritizes teamwork, participation, and consensus, and places great importance on group cohesion.	Constant	−2.733	0.596	20.99	<0.001	0.065	
	Clan culture	0.075	0.028	7.38	0.007	1.08	(1.02; 1.14)
	Hierarchical culture	0.090	0.030	8.97	0.003	1.09	(1.03; 1.16)
	Gender (1)	0.370	0.186	3.96	0.046	1.45	(1.01; 2.09)
C6. In Company B there is a strong concern for people.	Constant	−1.526	0.617	6.12	0.013	0.217	
	Clan culture	0.135	0.032	18.20	<0.001	1.15	(1.08; 1.22)

Obs. For readability, only statistically significant predictors are reported. All models included the same full set of predictors: Clan, Adhocracy, Hierarchy, Market, gender, age, residence, income, and employment status. Coding of categorical variables follows: Gender (1) Male; Employment (1) Employee.

**Table 7:** Adhocratic culture scenarios: Company choice between A and B.

Scenarios	Company A (High Salary)		Company B (As Described)	
	Frequency	%	Frequency	%
A1. Company B offers a dynamic, entrepreneurial and creative workplace.	181	31.0	403	69.0
A2. In Company B, innovation and risk-taking are accepted by employees and managers.	281	48.1	303	51.9
A3. In Company B, employees and managers are interested in experiencing everything new and appreciate different thinking.	220	37.7	364	62.3
A4. In Company B, the long-term focus is on growing the business and acquiring new resources.	285	48.8	299	51.2
A5. In Company B, success means achieving unique and new products or services, being a leader in the sector is very important.	317	54.3	267	45.7
A6. In Company B, individual initiative and freedom are encouraged.	161	27.6	423	72.4

**Table 8:** Adhocratic culture vs. compensation: Model fit Logistic regression results.

Scenario	Omnibus Test		Hosmer–Lemeshow		−2 Log Likel.	Cox & Snell R^2^	Nagelkerke R^2^	Class. Acc. (%)
	χ^2^ (df)	sig	χ^2^ (df)	sig				
A1. Company B offers a dynamic, entrepreneurial and creative workplace.	30.960 (15)	0.009	12.949 (8)	0.114	692.085	0.052	0.073	69.2
A2. In Company B, innovation and risk-taking are accepted by employees and managers.	31.831 (15)	0.007	5.730 (8)	0.677	776.936	0.053	0.071	60.8
A3. In Company B, employees and managers are interested in experiencing everything new and appreciate different thinking.	26.118 (15)	0.037	8.373 (8)	0.398	747.602	0.044	0.060	64.6
A4. In Company B, the long-term focus is on growing the business and acquiring new resources.	52.566 (15)	<0.001	10.399 (8)	0.238	756.694	0.086	0.115	62.0
A5. In Company B, success means achieving unique and new products or services, being a leader in the sector is very important.	48.878 (15)	<0.001	14.691 (8)	0.065	756.432	0.080	0.107	63.2
A6. In Company B, individual initiative and freedom are encouraged.	30.121 (15)	0.011	2.816 (8)	0.945	657.634	0.050	0.073	72.9

**Table 9:** Adhocratic culture vs. compensation: Logistic Regression Coefficients and Odds Ratios.

Scenario	Predictor	B	S.E.	Wald	*p*-Value	Exp(B)	95% CI for Exp(B)
A1. Company B offers a dynamic, entrepreneurial and creative workplace.	Constant	−1.529	0.593	6.65	0.010	0.217	
	Adhocratic culture	0.100	0.032	9.81	0.002	1.11	(1.04; 1.18)
	Employment (3)	0.868	0.430	4.08	0.043	2.38	(1.03; 5.53)
A2. In Company B, innovation and risk-taking are accepted by employees and managers.	Constant	−1.395	0.565	6.10	0.013	0.248	
	Adhocratic culture	0.076	0.030	6.44	0.011	1.08	(1.02; 1.14)
	Hierarchical culture	0.080	0.029	7.51	0.006	1.08	(1.02; 1.15)
	Market culture	−0.062	0.027	5.24	0.022	0.940	(0.89; 0.99)
A3. In Company B, employees and managers are interested in experiencing everything new and appreciate different thinking.	Constant	−1.352	0.573	5.56	0.018	0.259	
	Adhocratic culture	0.072	0.031	5.48	0.019	1.07	(1.01; 1.14)
	Employment (3)	0.740	0.386	3.68	0.055	2.10	(0.98; 4.47)
A4. In Company B, the long-term focus is on growing the business and acquiring new resources.	Constant	−2.369	0.586	16.32	<0.001	0.094	
	Hierarchical culture	0.103	0.030	12.03	<0.001	1.11	(1.05; 1.18)
	Income (1)	−0.524	0.267	3.85	0.050	0.592	(0.35; 1.00)
	Employment (3)	0.902	0.372	5.87	0.015	2.47	(1.19; 5.11)
A5. In Company B, success means achieving unique and new products or services, being a leader in the sector is very important.	Constant	−3.283	0.611	28.89	<0.001	0.038	
	Hierarchical culture	0.026	0.030	7.80	0.005	1.09	(1.02; 1.15)
	Income (3)	0.801	0.347	5.31	0.021	2.23	(1.28; 4.40)
A6. In Company B, individual initiative and freedom are encouraged.	Constant	−0.940	0.603	2.43	0.119	0.391	
	Adhocratic culture	0.074	0.033	5.04	0.025	1.08	(1.01; 1.15)
	Employment (3)	0.954	0.603	2.43	0.044	2.60	(1.02; 6.59)

Obs. For readability, only statistically significant predictors are reported. All models included the same full set of predictors: Clan, Adhocracy, Hierarchy, Market, gender, age, residence, income, and employment status. Coding of categorical variables follows: Employment (3) Never employed; Income (1) <1500 RON (~€300), (3) 2501–4000 RON (~€500–€800).

**Table 10:** Hierarchical culture scenarios: Company choice between A and B.

Scenarios	Company A (High Salary)		Company B (As Described)	
	Frequency	%	Frequency	%
H1. Company B offers a very structured and formal workplace where rules and procedures govern the behavior of employees and even managers.	322	56.8	252	43.2
H2. In Company B, managers strive to be good coordinators and organizers and are always oriented towards efficiency.	223	38.2	361	61.8
H3. In Company B, formal policies hold the group together.	339	58.0	245	42.0
H4. In Company B, stability, performance, and efficient operations are the long-term goals.	247	42.3	337	57.7
H5. In Company B, success means providing reliability, smooth scheduling, and low costs.	257	44.0	327	56.0
H6. In Company B, management wants security and predictability.	308	52.7	276	47.3

**Table 11:** Hierarchical culture vs. compensation: Model fit Logistic regression results.

Scenario	Omnibus Test		Hosmer–Lemeshow		−2 Log Likel.	Cox & Snell R^2^	Nagelkerke R^2^	Class. Acc. (%)
	χ^2^ (df)	sig	χ^2^ (df)	sig				
H1. Company B offers a very structured and formal workplace where rules and procedures govern the behavior of employees and even managers.	60.988 (15)	<0.001	7.497 (8)	0.484	737.614	0.099	0.133	65.9
H2. In Company B, managers strive to be good coordinators and organizers and are always oriented towards efficiency.	45.550 (15)	<0.001	8.682 (8)	0.370	731.126	0.075	0.102	64.6
H3. In Company B, formal policies hold the group together.	56.494 (15)	<0.001	11.197 (8)	0.191	737.906	0.092	0.124	64.4
H4. In Company B, stability, performance, and efficient operations are the long-term goals.	26.772 (15)	0.031	6.281 (8)	0.616	768.898	0.045	0.060	60.1
H5. In Company B, success means providing reliability, smooth scheduling, and low costs.	22.890 (15)	0.086	8.762 (8)	0.363	778.295	0.038	0.051	60.4
H6. In Company B, management wants security and predictability.	42.279 (15)	<0.001	5.079 (8)	0.749	765.562	0.070	0.093	60.8

**Table 12:** Hierarchical culture vs. compensation: Logistic Regression Coefficients and Odds Ratios.

Scenario	Predictor	B	S.E.	Wald	*p*-Value	Exp(B)	95% CI for Exp(B)
H1. Company B offers a very structured and formal workplace where rules and procedures govern the behavior of employees and even managers.	Constant	−2.814	0.603	21.81	<0.001	0.060	
	Hierarchical culture	0.176	0.032	29.57	<0.001	1.19	(1.12; 1.27)
H2. In Company B, managers strive to be good coordinators and organizers and are always oriented towards efficiency.	Constant	−1.668	0.579	8.30	0.004	0.189	
	Hierarchical culture	0.138	0.031	20.18	<0.001	1.15	(1.08; 1.22)
	Gender (1)	0.473	0.187	6.42	0.011	1.61	(1.11; 2.31)
H3. In Company B, formal policies hold the group together.	Constant	−2.844	0.605	22.09	<0.001	0.058	
	Adhocratic culture	−0.070	0.031	4.97	0.026	0.932	(0.88; 0.99)
	Hierarchical culture	0.148	0.032	21.76	<0.001	1.16	(1.09; 1.23)
H4. In Company B, stability, performance, and efficient operations are the long-term goals.	Constant	−1.460	0.565	6.68	0.010	0.232	
	Hierarchical culture	0.070	0.029	5.82	0.016	1.07	(1.01; 1.14)
	Gender (1)	0.426	0.181	5.54	0.019	1.53	(1.07; 2.18)
H5. In Company B, success means providing reliability, smooth scheduling, and low costs.	Constant	−1.376	0.562	6.00	0.014	0.253	
	Hierarchical culture	0.087	0.029	8.98	0.003	1.09	(1.03; 1.16)
H6. In Company B, management wants security and predictability.	Constant	−1.681	0.573	8.59	0.003	0.186	
	Hierarchical culture	0.149	0.031	23.37	<0.001	1.16	(1.09; 1.23)

Obs. For readability, only statistically significant predictors are reported. All models included the same full set of predictors: Clan, Adhocracy, Hierarchy, Market, gender, age, residence, income, and employment status. Coding of categorical variables follows: Gender (1) Male.

**Table 13:** Market culture scenarios: Company choice between A and B.

Scenarios	Company A (High Salary)		Company B (As Described)	
	Frequency	%	Frequency	%
M1. Company B is results-oriented and always focused on completing work.	284	48.6	300	51.4
M2. In Company B, people are competitive and goal-oriented.	299	51.2	285	48.8
M3. In Company B, managers are demanding, persistent, and productive.	287	49.1	297	50.9
M4. In Company B, the focus on winning unifies the organization, and reputation and success are shared concerns.	264	45.2	320	54.8
M5. In Company B, the long-term focus is on competitive action and achieving measurable goals and targets.	303	51.9	281	48.1
M6. In Company B, success means high market share and competitive prices.	334	57.2	250	42.8

**Table 14:** Market culture vs. compensation: Model fit Logistic regression results.

Scenario	Omnibus Test		Hosmer–Lemeshow		−2 Log Likel.	Cox & Snell R^2^	Nagelkerke R^2^	Class. Acc. (%)
	χ^2^ (df)	sig	χ^2^ (df)	sig				
M1. Company B is results-oriented and always focused on completing work.	43.690 (15)	<0.001	8.592 (8)	0.378	765.468	0.072	0.096	60.8
M2. In Company B, people are competitive and goal-oriented.	58.416 (15)	<0.001	8.062 (8)	0.427	750.845	0.095	0.127	64.4
M3. In Company B, managers are demanding, persistent, and productive.	58.526 (15)	<0.001	2.511 (8)	0.961	750.898	0.095	0.127	62.5
M4. In Company B, the focus on winning unifies the organization, and reputation and success are shared concerns.	39.401 (15)	<0.001	8.642 (8)	0.373	764.816	0.065	0.087	60.6
M5. In Company B, the long-term focus is on competitive action and achieving measurable goals and targets.	53.755 (15)	<0.001	6.863 (8)	0.551	755.011	0.088	0.119	63.2
M6. In Company B, success means high market share and competitive prices.	38.635 (15)	<0.001	7.183 (8)	0.517	758.837	0.064	0.086	62.3

**Table 15:** Market culture vs. compensation: Logistic Regression Coefficients and Odds Ratios.

Scenario	Predictor	B	S.E.	Wald	*p*-Value	Exp(B)	95% CI for Exp(B)
M1. Company B is results-oriented and always focused on completing work.	Constant	−1.822	0.574	10.08	0.001	0.162	
	Hierarchical culture	0.109	0.030	13.45	<0.001	1.12	(1.05; 1.18)
M2. In Company B, people are competitive and goal-oriented.	Constant	−2.418	0.585	17.09	<0.001	0.089	
	Adhocratic culture	−0.074	0.031	5.71	0.017	0.93	(0.87; 0.99)
	Hierarchical culture	0.129	0.031	17.78	<0.001	1.14	(1.07; 1.21)
M3. In Company B, managers are demanding, persistent, and productive.	Constant	−2.211	0.579	14.58	<0.001	0.110	
	Adhocratic culture	−0.069	0.031	4.96	0.026	0.93	(0.88; 0.99)
	Hierarchical culture	0.120	0.030	15.72	<0.001	1.13	(1.06; 1.20)
	Employment (1)	0.837	0.289	8.41	0.004	2.31	(1.31; 4.07)
	Employment (3)	0.736	0.360	4.17	0.041	2.09	(1.03; 4.23)
M4. In Company B, the focus on winning unifies the organization, and reputation and success are shared concerns.	Constant	−2.207	0.576	14.67	<0.001	0.110	
	Hierarchical culture	0.075	0.029	6.64	0.010	1.08	(1.02; 1.14)
	Gender (1)	0.379	0.182	4.36	0.037	1.46	(1.02; 2.09)
M5. In Company B, the long-term focus is on competitive action and achieving measurable goals and targets.	Constant	−2.378	0.586	16.46	<0.001	0.093	
	Hierarchical culture	0.135	0.031	19.44	<0.001	1.14	(1.08; 1.22)
M6. In Company B, success means high market share and competitive prices.	Constant	−1.768	0.573	9.51	0.002	0.171	
	Hierarchical culture	0.088	0.030	8.46	0.004	1.09	(1.03; 1.16)
	Market culture	0.055	0.027	4.10	0.043	1.06	(1.00; 1.16)
	Income (3)	0.739	0.346	4.56	0.033	2.09	(1.06; 4.13)

Obs. For readability, only statistically significant predictors are reported. All models included the same full set of predictors: Clan, Adhocracy, Hierarchy, Market, gender, age, residence, income, and employment status. Coding of categorical variables follows: Gender (1) Male, Employment (1) Employee, (3) Never employed; Income (3) 2501–4000 RON (~€500–€800).

## Data Availability

The raw data supporting the conclusions of this article will be made available by the authors on request.

## Abbreviations

The following abbreviations are used in this manuscript:

CVF	Competing Values Framework
OCAI	Organizational Culture Assessment Instrument
VIF	Variance Inflation Factor
OR	Odds Ratio
RON	Romanian New (Leu)
